# The Mediating Role of Parental Factors in the Social Patterning of Smoking among Adolescents in Urban Indonesia

**DOI:** 10.31557/APJCP.2021.22.10.3127

**Published:** 2021-10

**Authors:** Wahyu Septiono, Mirte A G Kuipers, Nawi Ng, Anton E Kunst

**Affiliations:** 1 *Department of Health Education and Behavioral Science, Faculty of Public Health, Universitas Indonesia. *; 2 *Public Health and Occupational Health, Amsterdam Public Health Research Institute, Amsterdam UMC, University of Amsterdam, Amsterdam, The Netherlands. *; 3 *School of Public Health and Community Medicine, Institution of Medicine, Sahlgrenska Academy, University of Gothenburg, Gothenburg, Sweden. *; 4 *Department of Epidemiology and Global Health, Faculty of Medicine, Umeå University, Umeå, Sweden. *

**Keywords:** Tobacco, smoking, adolescent, Indonesia

## Abstract

**Introduction:**

Parental factors may explain part of the social patterning of smoking among adolescents. This study aims at assessing the association between adolescent smoking and family characteristics (parental education, family wealth, and religion) and the mediating role of parental factors (smoking, control, and permissiveness towards smoking).

**Methods:**

In 2017, a cross-sectional survey was conducted in eight Indonesian cities among 2,393 students aged 13–18 years. Multilevel logistic regression analysis estimated the associations between family characteristics and adolescent smoking. Generalized Structural Equation Models (GSEM) quantified mediation of these associations by parental factors. Analyses were stratified by gender.

**Results:**

Smoking prevalence was 35.8% among boys and 2.6% among girls. Odds of smoking were higher among those with lower parental education among boys (low vs. high: OR:1.57, 95%CI:1.01-2.43), but not girls (OR:0.91, 95%CI:0.24-3.43). The association among boys was partially mediated by father’s smoking status, parental control, and parental permissiveness towards smoking. High family wealth was associated with higher odds of smoking among girls (poorer vs. wealthier: OR:0.39, 95%CI:0.15-0.99), but not boys (OR:0.76, 95%CI:0.52-1.10). This association among girls was not clearly mediated by parental factors. Religion was not associated with smoking among boys or girls.

**Conclusions:**

In Indonesia’s urban settings, inequalities in boys’ smoking by educational background may be addressed by measures aimed at supporting lower educated parents to improve parental control and to reduce permissiveness towards smoking.

## Introduction

The social patterning of smoking according to various demographic and socioeconomic indicators has been described in previous studies among adults (Casetta et al., 2016), and also among adolescents (Xi et al., 2016; Moor et al., 2019). For example, smoking prevalence is higher among adolescents in poorer families and whose parents are less educated (Bardach et al., 2016; Talip et al., 2016; Xi et al., 2016). Such inequalities have not only been found in high-income countries (Moor et al., 2019), but also in low and middle-income countries (LMICs) (Bardach et al., 2016; Talip et al., 2016; Xi et al., 2016; Kusumawardani et al., 2018).

Religious denomination has rarely been taken into account when describing the social patterning of adolescent smoking, despite previous research suggesting that cultural factors linked to religion may be an important determinant of adolescent smoking (Weaver et al., 2005; Barbosa Filho et al., 2012). Garrusi and Nakhaee (2012) state that various values in different religious communities may result in different social responses to smoking (Garrusi and Nakhaee, 2012). According to some Christian scholars, smoking may weaken the connection between them and God (Garrusi and Nakhaee, 2012). Among Hindus and Buddhists, smoking may violate a religious principle because it damages the body (Garrusi and Nakhaee, 2012). In Islam, smoking is often labelled as haram (strongly prohibited) or makruh (reprehensible but not fully prohibited) because smoking is not comprehensively referenced in two Islamic laws (i.e., Qur’an and Hadith) (Garrusi and Nakhaee, 2012).

Globally, Indonesia has a high smoking prevalence among boys aged 13-15, which increased from 24.5% in 2006 to 35.3% in 2014 (2006; World Health Organization Regional Office for South-East Asia, 2018). The prevalence among girls is low, but also increasing (from 2.4% in 2004 to 3.4% in 2014). There is limited evidence of socioeconomic inequalities in youth smoking in Indonesia, but one nationally representative study found that the odds of smoking were 2.5 times higher among adolescents in the lowest quintile of family wealth (Kusumawardani et al., 2018).

The majority of Indonesians are Muslim (88%) (Hefner, 2018), but a number of religions are recognised by law and practised in Indonesia (Barro and McCleary, 2003). The two biggest Muslim organisations in Indonesia have different views on smoking. Muhammadiyah, which has 30 million followers, states that smoking is haram (strongly prohibited), while Nahdlatul Ulama, with 40 million followers, declares that smoking is makruh (reprehensible) (Byron et al., 2015). To date, no official position about smoking has been released by Protestant, Catholic, Hindu, and Buddhist organisations in Indonesia.

It is important to understand which modifiable factors may cause the social patterning of adolescent smoking, so that future inequalities in adult smoking and smoking-related mortality may be decreased through interventions. The parent’s behaviours play an important role in adolescent smoking uptake (Thomas et al., 2007; Rosen et al., 2015). Parental smoking is associated with an increased risk of adolescent smoking uptake (Lochbuehler et al., 2016). Not only parents’ smoking behaviour, but also their permissiveness towards smoking may exert a more positive smoking norm and increase adolescent smoking (Voisine et al., 2008). A lack of parental control, in which parents are unaware of how and with whom their children spend their free time, has also been found to increase the likelihood of smoking, as peers may have a stronger negative influence on the adolescent (Osgood et al., 2005; Hiemstra et al., 2017). Moreover, limited parental control was found to mediate the association between adolescents being enrolled in a lower educational track and a higher risk of smoking (de Looze et al., 2012). These parental factors may explain part of the social patterning of smoking among adolescents in Indonesia, if parental practices are less favourable among adolescents with disadvantaged backgrounds. However, to our knowledge, the contribution of such parental factors to social patterning in adolescent smoking has not yet been studied in Indonesia.

In this study, we aimed to assess the mediating role of parental factors in the association between familial socio-demographic characteristics and adolescent smoking behaviour in Indonesia. Specifically, we studied (1) the association between family characteristics (i.e., parental education, family wealth and religion) and adolescent smoking, and (2) the extent to which parental factors (i.e., parental smoking, parental control, and parental permissiveness towards smoking) mediate the association between family characteristics and adolescent smoking. 

## Materials and Methods

*Data collection, study design, and participants*

During March-May 2017, a cross-sectional school-based survey was conducted among 2860 students aged 10 to 20 years old in 29 public schools in eight cities in Indonesia. The survey adapted questions from the European SILNE (Smoking Inequalities - Learning from Natural Experiments) survey (Kunst, 2016) and Indonesian National Socio-Economic Survey (SUSENAS) (Badan Pusat Statistik, 2017). Cities were selected purposively according to variation of local tobacco control policies (see Appendix Table A1) and similarity in GDP per-capita which ranged from 2406 – 3720 USD/person. In total, 22 non-religion-based public schools were invited and selected so as to achieve variation in geographical location within cities, based on distance to the City Hall. Seven schools were not willing to participate as pre-national examinations were in progress.

In grades 7 to 9 of junior high school and 10 to 12 of senior high school (typical age range 12 to 18 years), at least one class within each grade was randomly selected (i.e. minimum three per school, 134 in total). Teachers left the classroom during the survey. All participants provided informed consent and questionnaires were filled in anonymously. None of the students invited to the survey declined. Researchers explicitly communicated to all students that participation was voluntary. We excluded participants who were more than 18 years old and who did not answer questions on age (n=7), gender (n=10), smoking status (n=8), parents’ educational level (also ‘I don’t know’ responses) (n=107), wealth (n=30), religion (n=0), parental smoking status (n=0), parental control (n=6), parental permissiveness (n=299). In total we excluded 467 students resulting in a final analytical sample of 2393 students.

Ethical approval was obtained from the Indonesian Ministry of Health in March 2017 (LB.02.01/2/KE.097/2017). School principals’ permissions were obtained before the survey and parents were given information about the survey and contact of researchers. Trained enumerators performed the data collection. Two pilot surveys were conducted to assess and adapt the translated questionnaire.

*Measures*

*Dependent variable*

Smoking status was measured with the question: “How many cigarettes have you smoked during the last 30 days?”. Those who answered with “I have never smoked” and “none” were classified as non-smokers. Adolescents who smoked one cigarette or more in the last 30 days were categorised as current smokers.

*Family characteristics*

Adolescents reported their religion, parental education, family and wealth. Father’s and mother’s highest educational level were measured separately. We selected the educational level of the most highly educated parent, of which 38.5% adolescents reported high level (university graduate), 42.2% reported intermediate level (completed senior high school), and 19.3% reported low level (10.8% completed junior high school, 6.9% completed elementary school, 1.4% did not complete elementary school, and 0.2% did not attend school). 

Following the SUSENAS (Badan Pusat Statistik, 2017), which used the Family Affluence Scale (Boyce et al., 2006), wealth was measured using nine indicators: the family’s ownership (yes/no) of cars and motorcycles, type of energy source, own bedroom for respondent, radio, television, cell phone for respondent, computer, and refrigerator. We used principal component analysis (PCA) to calculate a wealth score which divided adolescents’ families into tertiles of poorer, intermediate, and wealthier. IBM SPSS version 25 was used to run PCA.

Adolescents indicated their religion from a list of five main religions that are officially recognised by Indonesian law. Reponses were categorised into three groups: Islam, Christianity (Catholic and Protestant), and Buddhism or Hinduism.

*Mediator variables*

Potential mediators were parental smoking, parental control, and parental permissiveness towards smoking. Parental smoking (yes/no) was reported by the adolescent for the father and the mother separately. Parents who did not cohabit with the respondent were considered non-smokers (n=13), as adolescents were assumed not to be exposed to their smoking.

Parental control was measured with three variables (1) “Do your parents pick you up from school?” (responses: ‘always’, ‘sometimes’, ‘never’) (2) “Do your parents ask about your activity outside school time?” (responses: ‘always’, ‘sometimes’, ‘never’) and (3) “Do your parents know the friends with whom you socialise?” (responses: ‘mostly’, ‘only a few’, ‘no’, ‘living without parents’). Participants who responded ‘living without parents’ to the third question were categorised into ‘no’ (n=13).

Parental permissiveness of smoking was measured with three variables (1) “Do your parents ask you to buy tobacco products for them?” (responses: ‘never’, ‘sometimes’, ‘always’) (2) “Is smoking permitted in your home (where you live all or most of the time)?” (responses: ‘no’, ‘yes, in certain areas’, ‘yes, everywhere’) and (3) “How would/do your parents react if they thought/knew you were smoking?” (responses: ‘disapprove a lot’, ‘disapprove a little’, ‘do not mind’, ‘approve’). Thirty-six participants who answered ‘approve’ to the third question were grouped together with ‘do not mind’.

*Statistical Analysis*

Multilevel logistic regression analysis, with adolescents clustered in schools and cities, was performed in Stata 14 (StataCorp, 2015). Adolescent smoking was the dependent variable in all analyses and all models were stratified by gender. In the age-adjusted model, we analysed all variables separately, only adjusting for age. Model 1 included all family characteristics variables (parental education, wealth and religion) and age. Model 2 additionally included all mediators. The intraclass correlation coefficient (ICC) was calculated in an ‘empty’ model that did not include any independent variables, and in both models. 

Next, a mediation analysis was performed using Generalised Structural Equation Modelling (GSEM) in Stata 14 (StataCorp, 2015). We estimated the contribution of eight mediators to the associations between family characteristics (parental education, wealth and religion, respectively) and smoking. The mediation analysis was conducted in multilevel logistic regression with all mediators included. For mediators with two categories (i.e., parental smoking variables), the indirect effect was calculated by multiplying the regression coefficients of the α and β paths (i.e., associations family characteristic–mediator and mediator–smoking, respectively; see Appendix Figure A1). For mediators with more than two categories (i.e., parental control and parental permissiveness), the indirect effect was calculated as the sum of indirect effects for each regression coefficient (i.e., ((α1×β1) + (α2×β2))). In the main tables, we presented only the indirect effects when the association between family characteristics and adolescent smoking was significant. Otherwise, the results of the mediation analysis were presented in the Appendix.

## Results

Table 1 displays the descdriptive analysis results for boys. The overall smoking prevalence among boys was 35.8% and was particularly high among boys whose parents did not mind them smoking (67.2%) or disapproved a little when they smoked (64.0%), whose mothers were smokers (53.3%), whose parents allowed smoking everywhere at home (51.9%), and who had poorly educated parents (51.3%). Prevalence was relatively low among boys who were always picked up by their parents after school (9.2%), boys younger than 15 years old (21.3%), and Christian boys (26.3%).

Table 1 also presents results of the regression analysis for boys. In Model 1, parental education showed an inverse relationship with smoking (low vs. high: OR:1.57, 95%CI:1.01-2.43). We did not find an association between wealth and smoking (poorer vs. wealthier: OR:0.76, 95%CI:0.52-1.10). Compared with Muslims, Christian boys (OR:0.77, 95%CI:0.46–1.30) and Hindu or Buddhist (OR:0.68, 95%CI:0.27-1.68) boys did not have significantly lower odds of smoking. In Model 2, boys whose fathers were smokers compared with boys whose fathers were non-smokers (OR:1.78, 95%CI:1.29-2.45) and boys who were never being picked up from school compared with boys who were always picked up (OR:5.36, 95%CI:2.80-10.24) had higher odds of smoking. The odds of smoking were higher when parents did not mind their son smoking (OR:4.00, 95%CI:2.21-7.23), or disapproved a little (OR:3.58, 95%CI:2.40-5.33), compared with those with strong disapproval.

Table 2 shows the descriptive analysis results for girls. Overall, smoking prevalence among girls was 2.6% and was higher among girls whose parents did not mind them smoking (22.2%), whose mothers were smokers (12.5%), who were always asked to buy cigarettes (11.8%), and whose parents never asked about their daily activities (10.5%). Prevalence was lower among girls who were always asked their daily activity (1.1%), who were younger than 15 years old (1.3%), and whose parents did not allow smoking at home (1.4%).

Table 2 also presents results of the regression analysis for girls. In Model 1, parental education was not associated with smoking among girls (moderate vs. high: OR:1.15, 95%CI:0.42-3.18; low vs. high: OR:0.91, 95%CI:0.24-3.43). Compared with girls living in wealthy families, the odds of smoking were lower in families of intermediate wealth (OR:0.34, 95%CI:0.13-0.89) and poorer wealth (OR:0.39, 95%CI:0.15-0.99). The odds of smoking were lower but not significant among Hindu/Buddhist girls than Muslim girls (OR:0.30, 95%CI:0.05-2.29). In Model 2, the odds of smoking were higher among girls whose mothers were smokers (OR:9.59; 95%CI:2.11-43.56), ‘never’ being asked about daily activity compared with ‘always’ (OR:5.78, 95%CI:1.39-23.99). Girls who were always asked to buy cigarettes (always vs. never: OR:6.49, 95%CI:2.09-20.16) and whose parents allowed smoking everywhere at home had higher odds of smoking (never allowed vs. allowed everywhere: OR:3.27; 95%CI:1.02-10.50). The odds of smoking were higher among girls whose parents did not strongly disapprove of smoking (OR:5.02, 95%CI 1.17-21.62), or did not mind about the girl’s smoking (OR:11.59, 95%CI 2.16-62.33), compared with those who disapproved a lot.

Table 3 shows the extent to which parental factors mediated the association between parental education and smoking among boys. Significant positive indirect effects were found for father’s smoking status (α×β intermediate compared to high: 0.336, 95%CI:0.088-0.585; α×β low compared to high: 0.527, 95%CI:0.160-0.894), picking up after school (α×β intermediate compared to high: 1.440, 95%CI: 0.033-2.847; α×β low compared to high: 4.190, 95%CI: 1.160-7.057) and reaction to smoking behaviour (α×β low compared to high: 1.730, 95%CI: 0.292-3.169). 

Table 4 presents mediation in the association between wealth and smoking. We did not observe any significant indirect effects of parental factors for girls. Hence the strong association between wealth and smoking among girls found in Table 2 was not mediated by the parental factors included in our analysis.

In Appendix Table A2, significant indirect effects in the association between parental education and smoking among girls were observed when smoking was allowed at home (α×β low compared to high: 2.679, 95%CI: 0.019-5.340) and reaction to smoking behaviour (α×β low compared to high: 5.063, 95%CI: 0.204-9.922). In Appendix Table A3, we did not observe any significant indirect effects in the association between wealth and smoking among boys. Appendix Table A4 estimates to what extent the association between religion and smoking was mediated by parental factors. Among boys, we did not observe any significant indirect effects of parental factors. The association between religion and adolescent smoking seemed to be mediated by parent asking their daughter to buy cigarettes, especially the difference in odds of smoking between Muslim and Buddhist/Hindu girls (α×β: -3.881, 95%CI=-7.637-0.126).

**Table 1:** Multilevel Logistic Regression of Factors Associated with Smoking among Adolescent Girls in Indonesia

		N	%^a^	Age-adjusted			Model 1			Model 2			
				OR		95% CI	OR		95% CI	OR		95% CI	
Total		1,139	35.8										
Age													
	10 - 14 years old	367	21.3	1.00			1.00			1.00			
	15 - 16 years old	438	41.6	2.00		(1.24 - 3.22)	1.95		(1.21 - 3.13)	1.61		(1.12 - 2.30)	
	17 - 18 years old	334	44.3	3.22		(1.87 - 5.54)	3.11		(1.81 - 5.35)	1.51		(1.02 - 2.22)	
Family characteristics													
Parental education													
	High	417	26.1	1.00			1.00			1.00			
	Moderate	498	37	1.04		(0.74 - 1.45)	1.10		(0.78 - 1.56)	1.20		(0.86 - 1.68)	
	Low	224	51.3	1.44		(0.94 - 2.20)	1.57		(1.01 - 2.43)	1.73		(1.15 - 2.61)	
Wealth													
	Wealthier	372	32.3	1.00			1.00			1.00			
	Intermediate	360	36.1	1.04		(0.74 - 1.48)	1.01		(0.71 - 1.43)	1.17		(0.82 - 1.66)	
	Poorer	407	38.8	0.81		(0.56 - 1.16)	0.76		(0.52 - 1.10)	0.95		(0.66 - 1.37)	
Religion													
	Muslim	950	37.1	1.00			1.00			1.00			
	Christian	98	26.5	0.77		(0.46 - 1.30)	0.78		(0.46 - 1.31)	0.77		(0.46 - 1.28)	
	Hindu and Buddhist	91	33	0.68		(0.27 - 1.68)	0.66		(0.26 - 1.62)	0.73		(0.39 - 1.38)	
Potential mediators													
Parental smoking													
	Father	675	42.4	1.81		(1.37 - 2.39)				1.78		(1.29 - 2.45)	
	Mother	45	53.3	1.85		(0.95 - 3.59)				1.72		(0.89 - 3.34)	
Parental control													
Picking up after school													
	Always	142	9.2	1.00						1.00			
	Sometimes	266	28.6	2.52		(1.28 - 4.94)				3.75		(1.91 - 7.35)	
	Never	731	43.6	3.41		(1.78 - 6.52)				5.36		(2.80 - 10.24)	
Asking daily activity													
	Always	475	31.6	1.00						1.00			
	Sometimes	592	38.2	1.08		(0.82 - 1.43)				1.09		(0.81 - 1.48)	
	Never	72	44.4	1.19		(0.68 - 2.09)				1.30		(0.72 - 2.35)	
Knowing peer group													
	Mostly	411	32.9	1.00						1.00			
	Only a few	561	36.7	1.05		(0.78 - 1.41)				0.91		(0.67 - 1.24)	
	No	167	40.1	1.08		(0.72 - 1.63)				0.97		(0.62 - 1.50)	
Parental permissiveness													
Asking to buy cigarettes													
	Never	711	33.6	1.00						1.00			
	Sometimes	277	39.4	1.19		(0.87 - 1.64)				0.84		(0.59 - 1.19)	
	Always	151	39.7	1.23		(0.83 - 1.83)				0.85		(0.56 - 1.29)	
Allowing smoking at home													
	Never	719	30.3	1.00						1.00			
	Yes, in certain areas	291	42.3	1.51		(1.10 - 2.05)				1.09		(0.74 - 1.41)	
	Yes, everywhere	129	51.9	1.67		(1.10 - 2.53)				1.01		(0.65 - 1.59)	
Parents' reaction if child smokes													
	Disapprove a lot	925	29.1	1.00						1.00			
	Disapprove a little	150	64	3.62		(2.43 - 5.38)				3.58		(2.40 - 5.33)	
	Do not mind	64	67.2	4.45		(2.45 - 8.11)				4.00		(2.21 - 7.23)	
City variance (SE)					0.447 (0.277)^b^			0.590 (0.231)			0.547 (0.206)	
City Intraclass correlation (%)					4.86^b^			8.83			7.93	
School variance (SE)					0.793 (0.176)^b^			0.555 (0.155)			0.422 (0.150)	
School Intraclass correlation (%)					20.11^b^			16.64			12.67	

^a^ Prevalence of smoking, in %, among the total population and within each subgroup.;^b^ Derived from an ‘empty’ model that did not include any independent variables.

**Table 2:** Multilevel Logistic Regression of Factors Associated with Smoking among Adolescent Boys in Indonesia

			N		%^a^		Age-adjusted			Model 1					Model 2		
							OR	95% CI		OR		95% CI			OR	95% CI	
Total			1,254		2.6												
Age																	
	10 - 14 years old		451		1.3		1.00			1.00					1.00		
	15 - 16 years old		446		3.4		3.57	(1.10 - 11.57)		4.21		(1.23 - 14.44)			3.23	(1.03 - 10.14)	
	17 - 18 years old		357		3.1		3.98	(1.01 - 15.56)		4.94		(1.15 - 21.13)			2.49	(0.71 - 8.80)	
Family characteristics																	
Parental education																	
	High		504		1.8		1.00			1.00					1.00		
	Moderate		513		2.9		1.04	(0.39 - 2.77)		1.15		(0.42 - 3.18)			1.59	(0.59 - 4.26)	
	Low		237		3.4		0.75	(0.21 - 2.64)		0.91		(0.24 - 3.43)			0.99	(0.27 - 3.17)	
Wealth																	
	Wealthier		431		3.5		1.00			1.00					1.00		
	Intermediate		430		1.6		0.35	(0.14 - 0.91)		0.34		(0.13 - 0.89)			0.39	(0.13 - 1.13)	
	Poorer		393		2.5		0.40	(0.16 - 1.01)		0.39		(0.15 - 0.99)			0.62	(0.23 - 1.68)	
Religion																	
	Muslim		1,038		2.7		1.00			1.00					1.00		
	Protestant and Catholic		93		2.2		0.92	(0.21 - 4.12)		0.89		(0.19 - 4.09)			0.86	(0.17 - 4.52)	
	Hindu and Buddhist		123		1.6		0.39	(0.06 - 2.50)		0.30		(0.05 - 2.29)			0.55	(0.05 - 5.98)	
Potential mediators																	
Parental smoking																	
	Father		658		2.7		1.01	(0.49 - 2.11)							0.42	(0.15 - 1.15)	
	Mother		32		12.5		8.54	(2.51 - 29.10)							9.59	(2.11 - 43.56)	
Parental control																	
Picking up after school																	
	Always		306		0.7		1.00								1.00		
	Sometimes		412		2.4		3.50	(0.74 - 16.49)							3.40	(0.68 - 17.01)	
	Never		536		3.7		4.47	(0.99 - 20.17)							3.44	(0.69 - 17.16)	
Asking daily activity																	
	Always		615		1.1		1.00								1.00		
	Sometimes		582		3.3		2.71	(1.11 - 6.59)							2.09	(0.79 - 5.58)	
	Never		57		10.5		9.68	(3.01 - 31.14)							5.78	(1.39 - 23.99)	
Knowing peer group																	
	Mostly		809		1.9		1.00								1.00		
	Only a few		384		3.4		1.71	(0.79 - 3.67)							1.49	(0.58 - 3.80)	
	No		61		6.6		3.08	(0.94 - 10.05)							0.98	(0.21 - 4.80)	
Parental permissiveness																		
Asking to buy cigarettes																		
	Never	804		1.6		1.00								1.00				
	Sometimes	348		2		1.18			(0.46 - 3.03)					1.34			(0.45 - 4.00)	
	Always	102		11.8		7.38			(3.12 - 17.48)					6.49			(2.09 - 20.16)	
Allowing smoking at home																		
	Never	583		1.4		1.00								1.00				
	Yes, in certain areas	486		3.1		2.14			(0.89 - 5.17)					2.44			(0.87 - 6.86)	
	Yes, everywhere	185		4.9		2.73			(0.98 - 7.58)					3.27			(1.02 - 10.50)	
Parents' reaction if child smokes																		
	Disapprove a lot	1,186		2		1.00								1.00				
	Disapprove a little	50		8		3.32			(1.00 - 11.04)					5.02			(1.17 - 21.62)	
	Do not mind	18		22.2		18.21			(4.78 - 69.36)					11.59			(2.16 - 62.33)	
City variance (SE)						0.001 (0.399) ^b^					0.001 (0.589)			0.001 (0.469)				
City Intraclass correlation (%)						0.01 ^b^					0.01			0.01				
School variance (SE)						0.805 (0.299) ^b^					1.072 (0.375)			0.921 (0.378)				
School Intraclass correlation (%)						16.46^b^					25.88			20.48				

**Table 3:** Mediation of the Associations between Parental Education and Smoking among Boys: Regression Coefficients and 95% Confidence Intervals for the Indirect Effect (i.e. α×β).

	Parental education			
	Intermediate compared to high		Low compared to high	
	α×β	95% CI	α×β	95% CI
Boys				
Parental smoking				
Father’s smoking status	0.336	0.088, 0.585	0.527	0.160, 0.894
Mother’s smoking status	0.440	-0.245, 1.125	0.198	-0.408, 0.804
Parental control				
Picking up after school	1.440	0.033, 2.847	4.109	1.160, 7.057
Asking daily activity	0.277	-0.354, 0.908	0.327	-0.409, 1.063
Knowing peer group	-0.011	-0.166, 0.144	-0.075	-0.544, 0.394
Parental permissiveness				
Asking to buy cigarettes	-0.179	-0.529, 0.172	-0.255	-0.757, 0.248
Allowing smoking at home	0.012	-0.381, 0.405	0.031	-0.750, 0.817
Reaction to smoking	0.748	-0.429, 1.925	1.730	0.292, 3.169

**Table 4:** Mediation of the Associations between Wealth and Smoking among Girls: Regression Coefficients and 95% Confidence Intervals for the Indirect Effect (i.e. α×β)

	Wealth			
	Intermediate compared to wealthier		Poorer compared to wealthier	
	α×β	95% CI	α×β	95% CI
Girls				
Parental smoking				
Father’s smoking status	-0.214	-0.561, 0.133	-0.256	-0.656, 0.143
Mother’s smoking status	-0.454	-2.265, 1.358	-2.878	-6.071, 0.316
Parental control				
Picking up after school	-1.443	-3.421, 0.535	-0.729	-2.023, 0.564
Asking daily activity	0.247	-1.063, 1.557	0.800	-0.745, 2.344
Knowing peer group	-0.045	-0.353, 0.263	-0.029	-0.956, 0.898
Parental permissiveness				
Asking to buy cigarettes	-0.569	-1.678, 0.539	0.464	-0.700, 1.628
Allowing smoking at home	0.067	-0.597, 0.732	0.122	-0.602, 0.846
Reaction to smoking	-0.907	-3.927, 2.114	-3.266	-7.605, 1.073

## Discussion

Smoking prevalence was 35.8% among boys and 2.6% among girls. Among boys, but not girls, smoking was inversely associated with parental education. This association was mediated by father’s smoking status, parental control (specifically whether parents pick their son up after school) and parental permissiveness (specifically disapproval of their child smoking). Girls in poorer families had lower odds of smoking, while wealth was not associated with smoking in boys. The association of wealth with girls’ smoking was not significantly mediated by parental factors. Among boys and girls, odds of smoking were lower but not significantly so among Christians and Buddhists/Hindus compared with Muslims. Part of the latter association may be mediated by whether girls were asked by their parents to buy cigarettes.

Some limitations need to be taken into account when interpreting the results of this study. First, the cross-sectional design implies that we should be careful not to draw any causal inference. We consider reverse causation in the associations of family characteristics and parental smoking with adolescent smoking as highly unlikely. However, we cannot rule out that parental control and permissiveness towards smoking may be influenced by the adolescent’s smoking status. Second, there may be residual confounding in the relationships with adolescent smoking. There may be risk factors that we did not control for, including anxiety or stress levels, or peer smoking status. Third, we measured the religion individuals adhered to, but not their religiosity level. Thus, participants may consider themselves part of a religious group, but may not necessarily practice their religion in daily life. Fourth, as the study was set in public schools in urban locations, our results may not generalise to rural settings, to private and religious schools, and to adolescents who do not receive formal education.

According to the Indonesian Global Youth Tobacco Survey (GYTS), the smoking prevalence among boys aged 13–15 in 2018 was 35.3% (World Health Organization Regional Office for South-East Asia, 2018), which is slightly higher than our estimates for boys in the same age group (27.0%). We found a smoking prevalence of 18.4% among 10 to 18 years old Indonesian adolescents (girls and boys combined). A much lower prevalence is observed in the 2013 national health survey (RISKESDAS), with a 6.6% prevalence rate for respondents in the same age group who lived in the same cities as our study sample (own calculations based on unpublished data described in (Ministry of Health, 2013)). Part of the discrepancy may be due to the use of different definitions of smoking in RISKESDAS compared with those used in the GYTS and our survey, as well as the use of different methods in the data collection. In the GYTS and our survey, a school-based survey was conducted, without the presence of teachers. RISKESDAS was a household survey in which respondents were interviewed in their homes, often in the presence of their parents, which may have led to strong underreporting due to desirability bias.

We found higher smoking prevalence among boys whose parents were less educated. An international study from Indonesia, Thailand, Taiwan, and the Philippines observed a weak association between parental education and smoking among boys (Kim Choe et al., 2004). In studies among South Korean and Chinese adolescents, a stronger gradient in adolescent smoking was found across parental education levels (Li et al., 2010; So and Yeo, 2015; Talip et al., 2016). We identified potential mediation by parental control, as indicated by poorly educated parents being less likely to pick their sons up from school (Ng et al., 2007). This is in line with a study from the Netherlands by De Looze et al., (2012) who found parental control to be a mediator in the association between adolescent educational level and smoking. Parents who are less in control are often less aware on their children whereabouts (de Looze et al., 2012). In Indonesia, highly educated parents often pick their children up after school to bring them to an afterschool course, while lower educated parents usually cannot afford afterschool courses (Mukminin et al., 2013). This may unintentionally increase unstructured time socialising with peers, during which smoking initiation often occurs (Osgood et al., 2005; de Looze et al., 2012). 

Family wealth had a strong negative association with smoking among girls, but not among boys. We did not observe mediation by parental factors in this association among girls, which suggests the direct effects of poor wealth on protecting the girls from being current smokers were more important than the indirect effects of other parental factors. The association may reflect a general social development in which smoking among Indonesian girls is more accepted and found within higher socioeconomic status (SES) groups. This also reflects that cigarettes were affordable for girls from wealthier families, as these girls often received more pocket money than girls from poorer families (Prabandari and Dewi, 2016). In Indonesia and other Asian countries, smoking is traditionally associated with men and masculinity (Barraclough, 1999). Poorer families are typically more conservative and traditional while wealthier families may generally be considered more progressive (Dagli et al., 2005). In several Asian countries, tobacco marketing has depicted female smoking as fashionable, modern, and Western, which may appeal to girls in wealthier communities (World Health Organization, 2010). This resembles the marketing of smoking as a symbol of Western freedom that was aimed at women in the former Soviet countries of central and Eastern Europe at the end of the 20th century (Amos and Haglund, 2000).

Smoking rates were consistently lower among Christians, Buddhists, and Hindus than among Muslims, although this was not statistically significant. Smoking is more frequent among Muslims possibly because it is not explicitly prohibited in the Koran and Sunna (i.e., the primary sources of Islamic law) (Ghouri et al., 2006). This may lead to different perceptions, though there is widespread tolerance to smoking among Muslims and the social norm towards smoking tends to be relatively positive. Hinduism and Buddhism, on the other hand, have a set of personal ethics as a main underlying principle (McDaniel, 2017), as opposed to laws, and both religions may be disapproving of smoking (Garrusi and Nakhaee, 2012). Smoking among girls may be more discouraged in Hindu and Buddhist families as these tend to be more traditional and therefore less susceptible to modern influences, including female-oriented tobacco advertisements. Involvement of religious leaders and religious schools in educational campaigns and other smoking prevention measures may be necessary to change smoking norms of Muslim parents and children.

In conclusion, we have identified groups of adolescents with an increased risk of smoking. Particular attention is warranted for boys of lower educational backgrounds, where smoking behaviour of fathers and less strict parental control may result in higher smoking rates. Preventive actions focusing on these parents may include sharing information regarding their influence on children’s smoking (educational campaigns, advice when contacting a health service), smoking cessation support programmes for lower educated parents, and actions to help them to increase monitoring (e.g. school-based services to monitor children outside school hours). 

## Author Contribution Statement

WS: funding acquisition, conceptualisation, development of methodology, data management and analysis, writing of draft and final versions of the paper, MAGK: conceptualisation, development of methodology, review and editing of paper versions, NN: advice on methodology, commenting on draft papers, AEK: conceptualisation, advice on methodology, commenting on draft papers.
